# Wildfires and social media discourse: exploring mental health and emotional wellbeing through Twitter

**DOI:** 10.3389/fpubh.2024.1349609

**Published:** 2024-04-12

**Authors:** Yury E. García, Miryam Elizabeth Villa-Pérez, Kuang Li, Xiao Hui Tai, Luis A. Trejo, Maria L. Daza-Torres, J. Cricelio Montesinos-López, Miriam Nuño

**Affiliations:** ^1^Department of Public Health Sciences, University of California, Davis, Davis, CA, United States; ^2^School of Engineering and Sciences, Tecnologico de Monterrey, Atizapán de Zaragoza, Mexico; ^3^Department of Statistics, University of California, Davis, Davis, CA, United States

**Keywords:** mental health, wildfire, X (a.k.a Twitter), wildfire emotional impact, sentiment analysis, LIWC, topic modeling, Tubbs Fire

## Abstract

**Introduction:**

The rise in global temperatures due to climate change has escalated the frequency and intensity of wildfires worldwide. Beyond their direct impact on physical health, these wildfires can significantly impact mental health. Conventional mental health studies predominantly rely on surveys, often constrained by limited sample sizes, high costs, and time constraints. As a result, there is an increasing interest in accessing social media data to study the effects of wildfires on mental health.

**Methods:**

In this study, we focused on Twitter users affected by the California Tubbs Fire in 2017 to extract data signals related to emotional well-being and mental health. Our analysis aimed to investigate tweets posted during the Tubbs Fire disaster to gain deeper insights into their impact on individuals. Data were collected from October 8 to October 31, 2017, encompassing the peak activity period. Various analytical methods were employed to explore word usage, sentiment, temporal patterns of word occurrence, and emerging topics associated with the unfolding crisis.

**Results:**

The findings show increased user engagement on wildfire-related Tweets, particularly during nighttime and early morning, especially at the onset of wildfire incidents. Subsequent exploration of emotional categories using Linguistic Inquiry and Word Count (LIWC) revealed a substantial presence of negative emotions at 43.0%, juxtaposed with simultaneous positivity in 23.1% of tweets. This dual emotional expression suggests a nuanced and complex landscape, unveiling concerns and community support within conversations. Stress concerns were notably expressed in 36.3% of the tweets. The main discussion topics were air quality, emotional exhaustion, and criticism of the president's response to the wildfire emergency.

**Discussion:**

Social media data, particularly the data collected from Twitter during wildfires, provides an opportunity to evaluate the psychological impact on affected communities immediately. This data can be used by public health authorities to launch targeted media campaigns in areas and hours where users are more active. Such campaigns can raise awareness about mental health during disasters and connect individuals with relevant resources. The effectiveness of these campaigns can be enhanced by tailoring outreach efforts based on prevalent issues highlighted by users. This ensures that individuals receive prompt support and mitigates the psychological impacts of wildfire disasters.

## 1 Introduction

Extreme environmental events such as wildfires have become more frequent and intense, exposing millions of people to traumatic stressors and adverse living conditions (1). Several factors influence wildfire risk, including temperature, soil moisture, and potential fuel sources such as trees and shrubs. However, climate change has emerged as a significant driver in escalating the likelihood and spread of wildfires by intensifying the drying of organic forest materials (2). In this regard, climate change is a global challenge that severely threatens human health and wellbeing, particularly in California, where it is the main factor in increasing wildfire frequency and intensity, as higher temperatures and drier conditions are expected to cause fires to be more severe each year. In addition, changes in precipitation patterns, such as compressing winter precipitation into a shorter timeframe, are linked to a more extended fire season (3).

Wildfires cause severe and lasting damage worldwide. In 2020, the U.S. alone saw more than 10 million acres of land burned by 58,258 fires (4), with California experiencing an eight-fold increase in fire activity since the 1970s–2018 (5). These trends match California's 34.5°*F* temperature rise, mainly caused by human activities and exceeding global warming projections (1). California experienced its 15 worst wildfires in the last ten years, exposing 27 times more people to harmful particulate matter (PM2.5) and air pollutants (6). Particulate matter refers to tiny solid particles and liquid droplets suspended in the air, and PM2.5 specifically denotes particles with a diameter of 2.5 micrometers or smaller. Wildfires will likely worsen, exposing more people to harmful smoke. By 2100, half of the yearly PM2.5 pollution in the U.S. will be driven by wildfires, resulting in 44,000 excess deaths from smoke exposure every year (7).

California suffered devastating wildfires in 2017 and 2018, burning over 3.5 million acres of land, damaging over 33,000 structures, and causing nearly 300 deaths and injuries. The total economic loss was estimated at over *$*32 billion in 2021 (8). One of the worst wildfires (Tubbs Fire) in California originated near Calistoga on October 8, 2017. The fire quickly spread to Napa, Sonoma, and Lake counties. It burned about 37,000 acres, killing at least 22 people and destroying parts of Santa Rosa (9). Strong winds and dry conditions fueled the fire and it was part of a larger firestorm that affected eight counties in Northern California (10). The Tubbs Fire received widespread media attention and social media traffic on Twitter, especially on October 11, its most intense period (10). The Tubbs Fire was the most destructive fire in California history until the Camp Fire in 2018 (10, 11).

Wildfires can have a significant impact on people, physically and mentally. The consequences can be long-lasting and affect individuals in various ways. Survivors of wildfires often experience enduring effects on their mental wellbeing (12–14). Research indicates that most survivors endure life-threatening situations during these disasters, resulting in a heightened prevalence of conditions like depression and post-traumatic stress disorder (PTSD) in the aftermath (13, 15). The severity of exposure and additional traumas can exacerbate the risk of mental health disorders, including PTSD, depression, anxiety, and substance abuse (13, 16). Notably, a study by (17) observed that firefighters in Greece developed symptoms of PTSD within just a month of wildfire exposure. This highlights the severity of the impact that wildfires have on mental health, even for those who are trained to handle such situations. Furthermore, research findings on the repercussions of wildfires in California underscore significant and enduring mental health needs, particularly concerning access to professional psychological treatment (18, 19). It is important to emphasize that the repercussions of wildfire-related exposure extend beyond the immediate geographical areas directly affected. This phenomenon has been substantiated in reports by Eisenman and Galway (20) and corroborated by various other sources (21, 22).

Survey methods are commonly employed to study mental health in larger communities during wildfires (23–25). An illustrative case of addressing the mental health needs of individuals affected by disasters such as wildfires is using the Behavioral Risk Factor Surveillance System (BRFSS), a telephone survey conducted by the US Centers for Disease Control and Prevention (CDC) (26). However, despite its usefulness, this approach faces inherent limitations, including limited sample sizes, scalability, cost, a limited capacity to study various mental health conditions, and an inability to offer real-time monitoring of mental health trends, given the study design.

Analysis of the messages posted on social media platforms may provide insights into the emotional wellbeing of the population (19, 27). Hence, this study uses social media data as a source of information to investigate mental wellbeing in the context of wildfire exposure. Our main focus centers on Twitter, a platform where users share tweets to express their thoughts and emotions in response to various life events, including crises such as wildfires (23, 28, 29). We work under the hypothesis that during wildfire events, such as the Tubbs Fire, the negativity of sentiment expressed in users' tweets increases, reflecting the adverse emotional impact caused by the disaster. Furthermore, the topics of discussion on Twitter during this time are primarily focused on the loss, fear, and distress associated with wildfire, suggesting a relationship between traumatic events and the emotional content of online interactions. By leveraging social media data platforms, researchers can gain additional insights into the experiences and perceptions of individuals affected by wildfires, which can inform efforts to address the mental health needs of survivors.

The main objective of this research is to identify the linguistic and behavioral patterns of Twitter users during the occurrence of wildfires. In particular, we focus on analyzing the data collected during the Tubbs Fire in 2017, well-known for its devastating impact on California's history, causing widespread destruction and deep emotional trauma in affected communities. Using user tweets as a data source, we aim to assess the predominant sentiment in the community and explore the main topics of discussion during this critical period. In addition, we examine the disaster's effects on users' emotional state, providing insights into the possible psychological impacts of wildfires.

## 2 Methods

**Figure 1:**
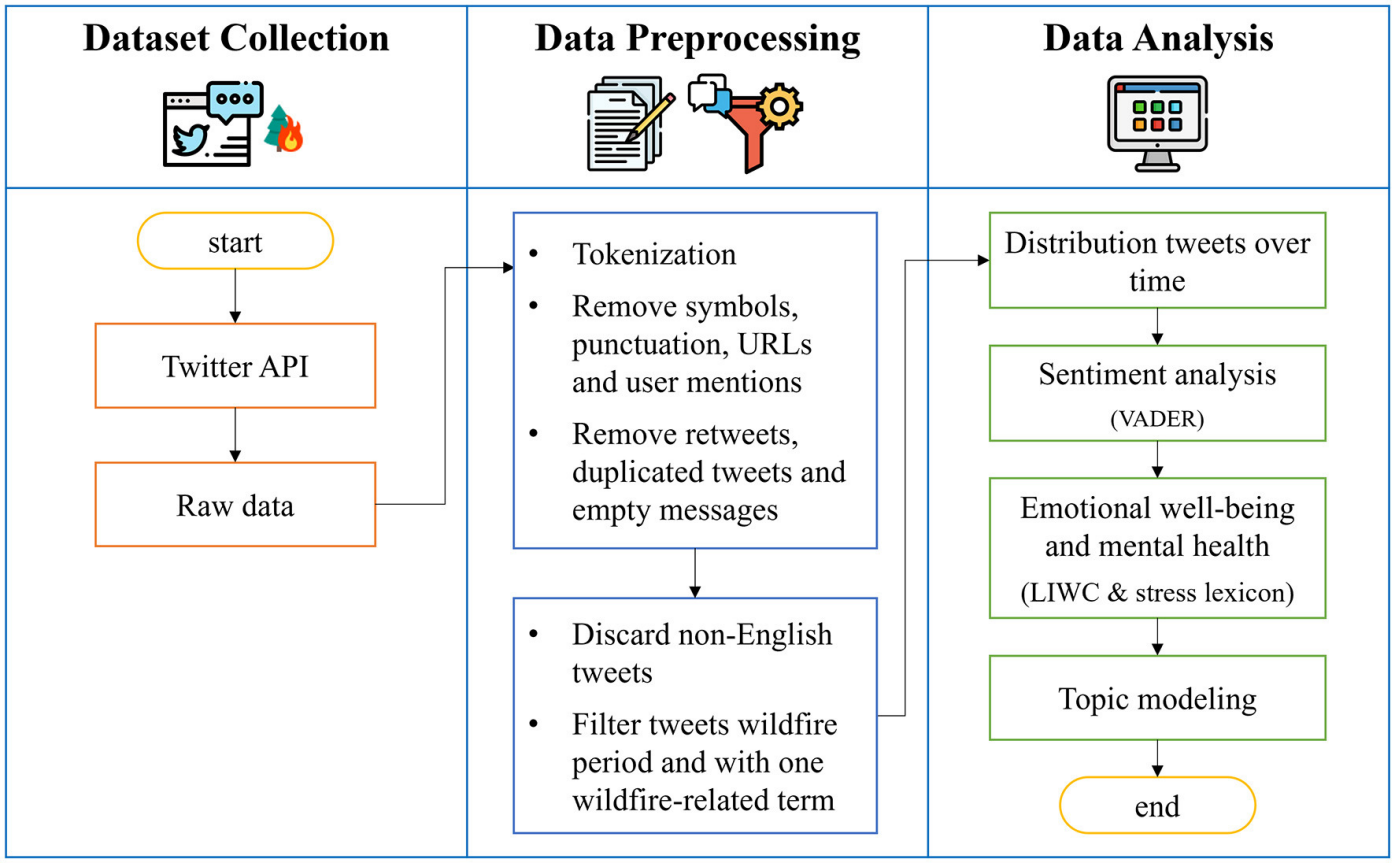
Process diagram of the study.

This section contextualizes the Tubbs Fire and discusses its impact and significance within the California wildfire landscape. Next, it provides a detailed description of the collected dataset, followed by a discussion of the methodologies used to explore the emotional tones expressed in the tweets and gain insights into the psychological aspects of the discourse. Finally, it outlines the topic modeling technique employed to identify and categorize the themes and discussions surrounding the Tubbs Fire. The process is shown in Figure 1.

The source code for our experiments is available in a GitHub repository,^1^ and the Tubbs Fire dataset is available for download through the IEEE DataPort platform.^2^

### 2.1 Tubbs fire context

The Tubbs Fire was a devastating wildfire in Northern California in October 2017. It became the most destructive wildfire in California's history, causing extensive damage in Napa, Sonoma, and Lake counties, with Santa Rosa being particularly hard hit. Its record was surpassed by the 2018 Camp Fire the following year (30). The Tubbs Fire was part of a series of large fires that ignited simultaneously in eight Northern California counties, known as the “Northern California firestorm” (10, 31).

By the time it was contained on October 31, the Tubbs Fire had scorched approximately 36,810 acres of land and tragically claimed the lives of at least 22 people in Sonoma County. The fire originated near Tubbs Lane in the northern rural area of Calistoga, Napa County. It destroyed more than 5,643 structures, including half of the homes in Santa Rosa (32). The economic loss in Santa Rosa alone was estimated to be around $1.2 billion in 2017 USD, with a five percent reduction in the city's housing inventory (10, 33).

**Figure 2:**
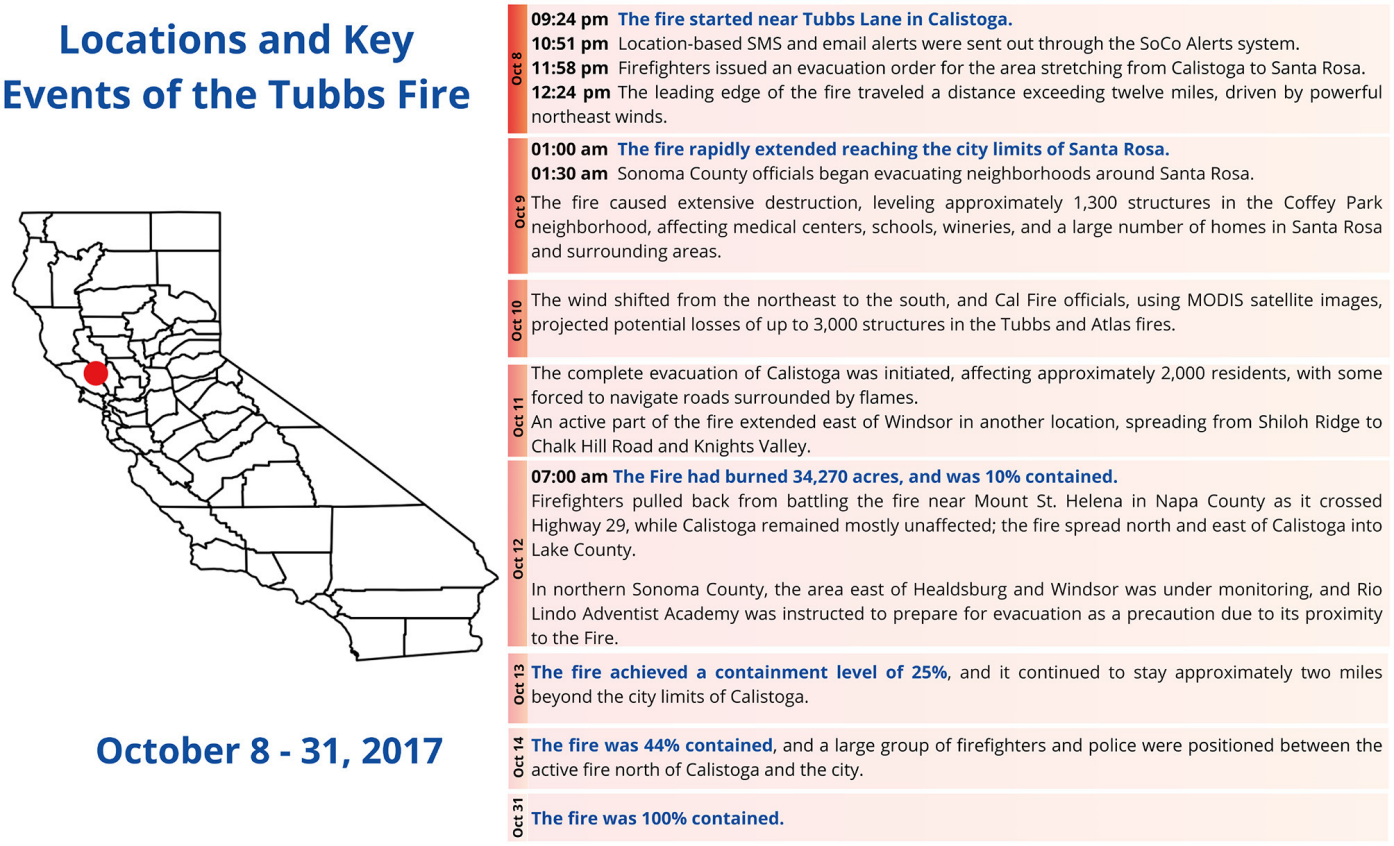
Summary of key events during the Tubbs Fire and its geographical location.

Figure 2 describes the principal events during the Tubbs Fire. The most crucial containment phase took place within the initial seven days, reaching a wildfire containment of approximately 50% by October 14. Subsequently, on October 18, significant progress became apparent in containment efforts, ultimately resulting in the successful containment of 92% of the fire. Finally, the wildfire was completely contained on October 31st (10).

### 2.2 Data collection and preprocessing

**Figure 3:**
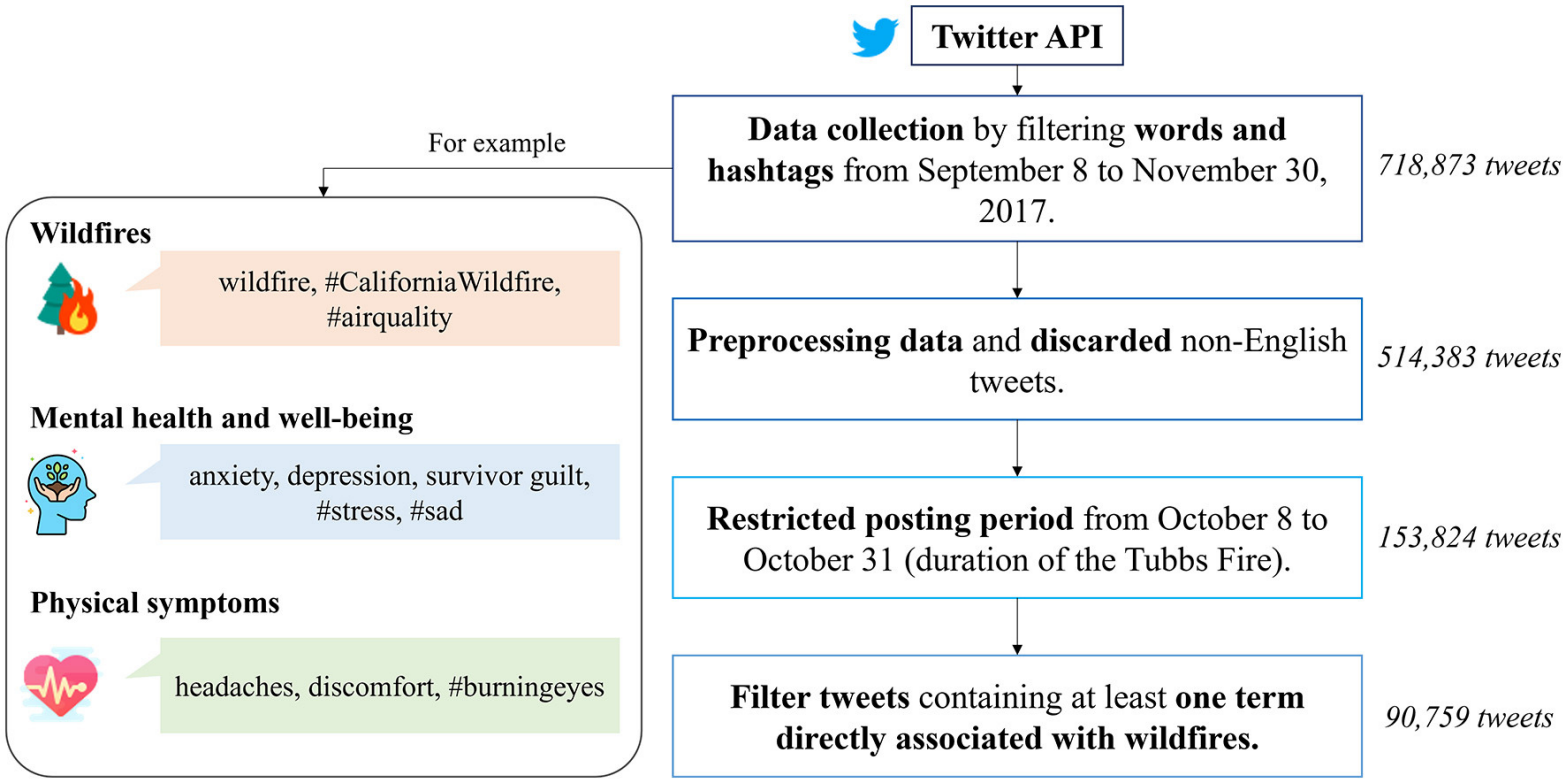
Overview of the data collection pipeline.

Data collection focused primarily on terms related to wildfire, mental health, wellbeing, physical symptoms associated with smoke and wildfire exposure, and hashtags commonly used during the Tubbs Fire (the complete list of used keywords is included in the Supplementary Material). All tweets obtained are public and gathered from Twitter between September 8 and November 30, 2017, through its application programming interface (API); the initial data consisted of 718,873 tweets. The selection of the tweets was computed using the full-archive endpoint provided by the Twitter API, which allows developers to request historical tweets by searching them via a set of rules or specific vocabulary and rapidly retrieve large-scale tweets. An overview of the data-collection pipeline is shown in Figure 3.

During the data cleaning process, we systematically removed symbols, special characters, punctuation, URLs, retweets, duplicated tweets, empty messages, those containing NaN values, and text that solely consisted of dates. Messages in languages other than English were also filtered out from the dataset. Furthermore, adhering to established natural language processing (NLP) practices, we eliminated stop words and performed tokenization. However, to perform sentiment analysis, we retained words associated with negations as they play a role in understanding the emotions and expressed sentiments. Following the data refinement steps, the dataset was reduced to 514,383 tweets.

Initially, data collection extended beyond the timeframe of the Tubbs Fire event. However, during the analysis phase, we focused exclusively on the period from October 8 to October 31, aligning precisely with the duration of the Tubbs Fire. As a result, the dataset was refined to include only tweets from this particular timeframe, yielding a total of 153,824 tweets available for analysis.

We strategically selected tweets based on specific criteria in the initial data-gathering phase. These criteria included wildfire-related keywords, mental health, and physical symptoms. This approach was designed to focus on wildfires and provide a comprehensive view of mental health and wellbeing indicators in the context of wildfire events. However, this method resulted in a dataset that included tweets associated only with mental or physical symptoms, not wildfire-related. Consequently, after data cleaning, we implemented a filtering mechanism to refine our dataset. This mechanism retained only those tweets that contained at least one term directly associated with wildfires, such as smoke, fire, or wildfires. Hence, the final dataset available for analysis comprises 90,759 tweets. This approach was utilized to create a dataset centered on discussions on a wildfire incident. Nevertheless, it is essential to acknowledge that multiple concurrent wildfires were effective during our analysis (10, 31), which may have led to the inclusion of data not solely related to the Tubbs Fire but encompassing broader wildfire incidents.

### 2.3 Sentiment analysis

Sentiment analysis, also referred to as opinion mining, is a computational methodology within the field of NLP and machine learning (34, 35). Its primary objective is to evaluate the underlying emotions conveyed in a text.

Sentiment analysis methods are broadly categorized into Lexicon-based and Machine Learning (ML)-based approaches. Lexicon-based methods can be further classified into dictionary-based techniques and corpus-based methods. These approaches utilize predefined dictionaries to evaluate sentiment based on positive, negative, or neutral words, or utilize statistical models derived from extensive text datasets to understand contextual nuances. ML-based methods, on the other hand, are divided into Supervised and Unsupervised Learning. Supervised Learning utilizes labeled data to train classification models such as SVM, KNN, DTC, and LR. In contrast, Unsupervised Learning uncovers patterns within data through clustering, topic modeling, and mapping algorithms (36).

In this analysis, we use sentiment analysis to identify and classify the predominant emotional tone expressed through the language used in each tweet. For the analysis, we use VADER (Valence Aware Dictionary and sentiment Reasoner) (37), a lexicon-based tool tailored for social media texts, available in the Python library called vaderSentiment.^3^

VADER relies on a lexicon, a predetermined list of words categorized with sentiment scores. Each word in this lexicon is assigned either a positive, negative, or neutral sentiment score, with some words receiving scores that denote the intensity of the sentiment they convey. When VADER encounters a word within a text, it cross-references it with its lexicon. If the word is found, the algorithm assigns the associated sentiment score to that word. For example, the word “happy” may receive a positive score, whereas “sad” might obtain a negative score.

VADER also incorporates negations and intensifiers in its sentiment analysis. For instance, when it comes across the expression “not happy”, it recognizes that the negation “not” inverts the sentiment of “happy” to negative. Additionally, it considers intensity modifiers like “very” or “extremely”, which enhance the sentiment. For instance, “very happy” would receive a higher positive score than simply “happy”. VADER amalgamates these individual word scores to calculate an overarching sentiment score for the entire sentence while also considering the sentence's structural context. It recognizes that phrases like “I'm not very happy” are less negative than “I'm extremely unhappy”. VADER also identifies essentially neutral words and refrains from assigning them strong positive or negative sentiment scores.

### 2.4 Linguistic inquiry and word count

Linguistic Inquiry and Word Count (LIWC) is a computational technique initially introduced for the analysis of text data (38, 39). It is well-known for its psychometric language analysis capabilities and remarkable effectiveness in unveiling language patterns associated with various disorders (40–42). This study used the LIWC2015 method to evaluate users' tweets for indicators of emotional wellbeing. LIWC analyzes text by identifying specific words from pre-defined categories in its library. These words are organized hierarchically into groups, including positive and negative emotions like happiness, sadness, anger, and anxiety. Some words might fall into broader categories, making it possible for a word to appear in multiple emotion categories, and its results show the percentage of words in each category within the text. LIWC emotion categories can accurately assess emotional expressions on Twitter (23, 40, 43). Unlike conventional sentiment analysis, LIWC delves deeper into the text's psychological and cognitive dimensions, providing insights into personality traits, emotional states, cognitive processes, social orientation, and other pertinent psychological attributes. Our analysis focuses on six emotional categories: anxiety, anger, sadness, health, and positive and negative emotions, as outlined in the LIWC library (39). Additionally, a dictionary on stress as described in Wang et al. (44) is incorporated.

### 2.5 Topic modeling

During the wildfire event, the Latent Dirichlet Allocation (LDA) algorithm and LIWC were used to identify the main topics discussed on Twitter and the most frequently used words to convey thoughts and emotions. LDA is a probabilistic generative model employed to find hidden topics within a corpus of textual documents, such as books or articles (45). In this research, LDA is adapted to unveil latent topics within a dataset of tweets (denoted as Z). The model achieves this task by probabilistically estimating critical parameters, namely alpha (α), beta (β), and theta (θ), utilizing distribution-based computations derived from the textual content.


(1) $$\begin{array}{l} p\left(Z,proportions,assignments|D\right)=p\left(\beta ,z,\theta |\omega \right) \end{array}$$

Equation 1 represents the joint probability distribution over the latent variable *Z* (the topic assignments for each word in the documents), proportions (θ, topic proportions for each document), and assignments (*z* refers to the specific assignment of words to topics in the documents) given the observed data D, which are the words in the documents (this is represented by ω in the equation). The model parameter β corresponds to the distribution of words for each topic. Hence, Equation 1 states that the probability of the topic assignments, proportions, and assignments given the observed data (the words in the documents) is equal to the joint probability *p*(β, *z*, θ|ω) of the model parameters and latent variables given the observed data. This is what we aim to maximize during the model fitting process.

The goal of LDA is to infer the latent variables (Z, β, and θ) that best explain the observed data (ω). This is typically done using iterative algorithms like variational inference or Gibbs sampling (45, 46).

The choice of the number of topics (K) is a hyperparameter of the LDA model and needs to be set before model fitting. It is often chosen based on domain knowledge or through model selection techniques. Also, LDA assumes that the order of the words in the document does not matter (bag-of-words assumption), which might not hold true for all types of text data (28). For *N* words in a document (*d*), θ is selected from Dirichlet (α). For each word (*w*_*n*_), the topic (*z*_*n*_) of the word is chosen from multinomial (θ) and a word (*w*_*n*_) from *p*(*w*_*n*_|*z*_*n*_, β).


(2) $$\begin{array}{l} p\left(\omega |\alpha ,\beta \right)=\int p\left(\theta |\alpha \right)\left(\overset{N}{\underset{n=1}{\prod }}\underset{z_{n}}{\sum }p\left(z_{n}|\theta \right)\cdot p\left(\omega_{n}|z_{n},\beta \right)\right)d\theta \end{array}$$

Equation 2 calculates the marginal probability of a document, and Equation 3 extends this to calculate the marginal probability of a corpus (a collection of documents), where M represents the total number of documents in the corpus.


(3) $$\begin{array}{l} p\left(D|\alpha ,\beta \right)=\overset{M}{\underset{d=1}{\prod }}\int p\left(\theta_{d}|\alpha \right)\left(\overset{N_{d}}{\underset{n=1}{\prod }}\underset{z_{d_{n}}}{\sum }p\left(z_{d_{n}}|\theta_{d}\right)p\left(\omega_{d_{n}}|z_{d_{n}},\beta \right)\right)d\theta_{d} \end{array}$$

We used Optuna (47), an optimization framework, for hyperparameter optimization to determine the best values for α, β, and the number of topics. The objective of the optimization problem is to maximize the coherence score, which is a metric used to assess the quality of an LDA model. We conducted a total of 50 trials to systematically explore the hyperparameter space to identify configurations that lead to improved topic quality. The hyperparameters encompass number of topics, recommended within the range of 10–100. Additionally, alpha, influencing document-topic density, was chosen between “symmetric” and “asymmetric” categorical choices. Lastly, the beta parameter influencing topic-word density, was suggested within a float range from 0.01 to 1.0.

## 3 Results

In this section, we present the results of the temporal distribution of tweets and sentiment, showing patterns and variations throughout the Tubbs Fire period. Furthermore, we examine the emotional content in the tweets, providing insights into the prevalent sentiments expressed throughout the wildfire event. Additionally, we discuss the most relevant topics that emerged within the discourse surrounding the Tubbs Fire, highlighting Twitter users' primary themes and concerns.

### 3.1 Distribution of tweets and sentiment over time

**Figure 4:**
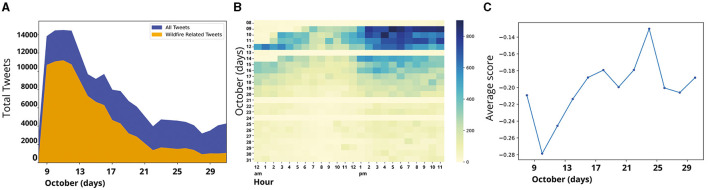
**(A)** Number of tweets over time. The blue region represents the total daily volume of initially downloaded tweets. The orange region shows the volume of tweets after filtering for terms like ‘wildfire' or ‘smoke'. **(B)** Heatmap of daily hourly tweet distribution. **(C)** Daily average sentiment scores.

Figure 4A illustrates the daily tweets collected from October 8 to October 31, 2017. The region in blue corresponds to daily cumulative tweets captured in the first set of downloaded data (153,824). In contrast, daily cumulative Tweets containing terms associated with “wildfire” or “smoke” (90,759) are shown in orange. During the initial week, Twitter users showed the most significant interest in the Tubbs Fire, especially as numerous communities were directly impacted by its rapid spread, resulting in devastating consequences in the early stages of the fire (10). However, as time advanced and substantial headway was achieved in containing the fire, reaching approximately 50% containment by October 14, a discernible decrease in Twitter activity emerged. This temporal correlation aligns with the observed decline in interest, reflecting the diminishing novelty and urgency of the event. Figure 4B displays the aggregation of tweets on an hourly and daily basis. This pattern aligns with the observations in Figure 4A, indicating that the most significant surge in Twitter activity is concentrated in the first days of the fire event. The findings suggest that Twitter activity peaks during the evening and nighttime.

The process of acquiring tweets involves starting from the final hour of the previous day and moving backwards in time. Tweets are collected in this reverse chronological order until the desired number of tweets is obtained. This method ensures that the most recent tweets are prioritized in the collection process. This approach resulted in gaps in the data; the algorithm prioritized completing tweet counts before covering all hours of the day. This phenomenon led to data omissions on October 13, 21, and 24. Figure 4A showcases smoothed data to mitigate the impact of such gaps, though they remain evident in the heat-map representation.

Regarding the sentiment analysis over time, the graph in Figure 4C portrays daily average sentiment scores, revealing a noticeable trend. Mainly, there is a decline in sentiment scores during the initial days depicted in the graph, right after the onset of the wildfire. This significant decrease in sentiment corresponds to the fire's immediate aftermath, reflecting the emotional toll on the community (negative values indicate negative emotions).

### 3.2 Emotional content within the tweets

**Figure 5:**
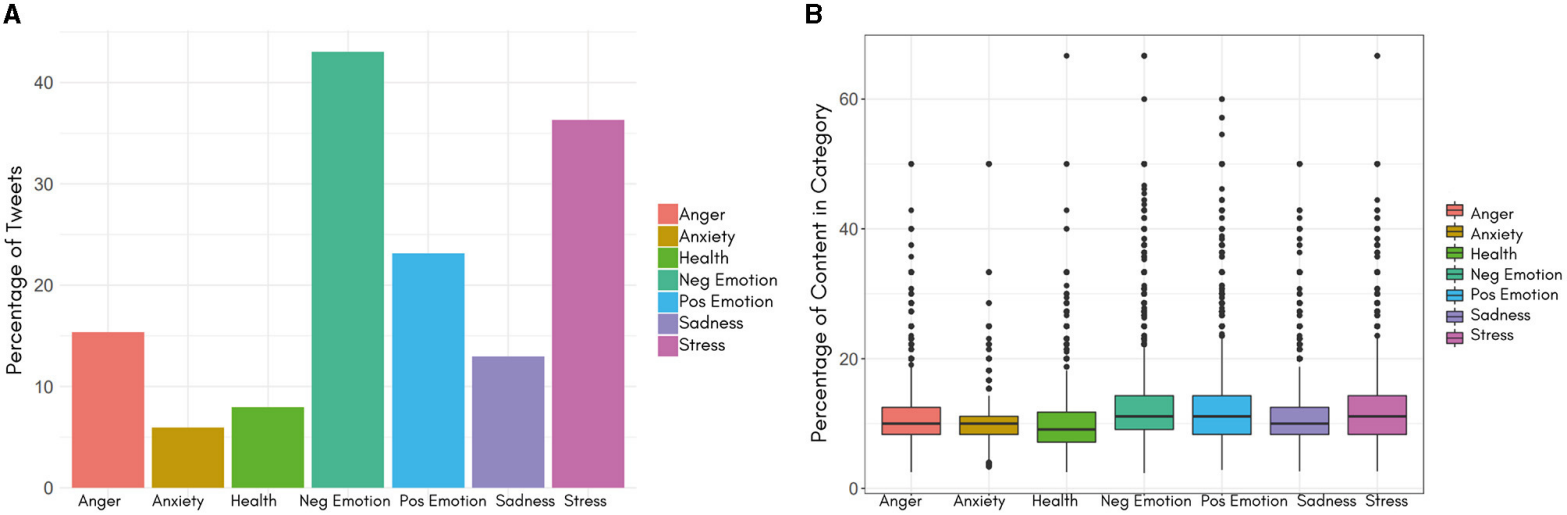
**(A)** Distribution of tweets among various emotional categories (a single tweet can simultaneously fall into multiple categories). **(B)** Boxplot showing central tendency, variability, and possible outliers in the content percentage within each tweet for each category.

LIWC provides insights into the frequency or proportion of words within a given tweet that align with specific linguistic and emotional categories. The resultant boxplot (Figure 5B) and the Table 2 showcase tweets from various categories. Summarized statistics detailed in Table 1 collectively provide a comprehensive portrait of the distribution and attributes of content associated with distinct emotional categories within the examined dataset. The boxplot visually conveys the central tendency, spread, and potential outliers for each category, providing an understanding of the spectrum of emotional expressions present in the tweets.

**Table 1:** Summary of LIWC category statistics in analyzed tweets.

**Category**	**Mean**	**Median**	**SD**	**Min**	**Max**	**Total Tweets**	**%**
Anger	11.2	10.0	4.4	2.5	50.0	13,911	15.3
Anxiety	10.1	10.0	3.5	3.3	50.0	5,354	5.9
Health	10.3	9.1	4.2	2.5	66.7	7,190	7.9
Neg emotion	12.8	11.1	5.8	2.4	66.7	39,018	43.0
Pos emotion	12.7	11.1	6.2	2.9	60.0	20,994	23.1
Sadness	11.3	10.0	4.7	2.6	50.0	11,731	12.9
Stress	12.2	11.1	5.3	2.6	66.7	32,926	36.3

Table 1 shows that the “Neg Emotion” category stands out with the highest mean and median values, recording 12.8 and 11.1, respectively, and a standard deviation of 5.8. These figures highlight the predominance of negative emotional content in the analyzed tweets. Similarly, the “Pos Emotion” and “Stress” categories exhibit mean and median values of 12.7 and 11.1, and 12.2 and 11.1, respectively. These findings suggest the coexistence of positive emotional expressions and discussions related to stress, highlighting the multifaceted nature of responses to the wildfire event.

**Figure 6:**
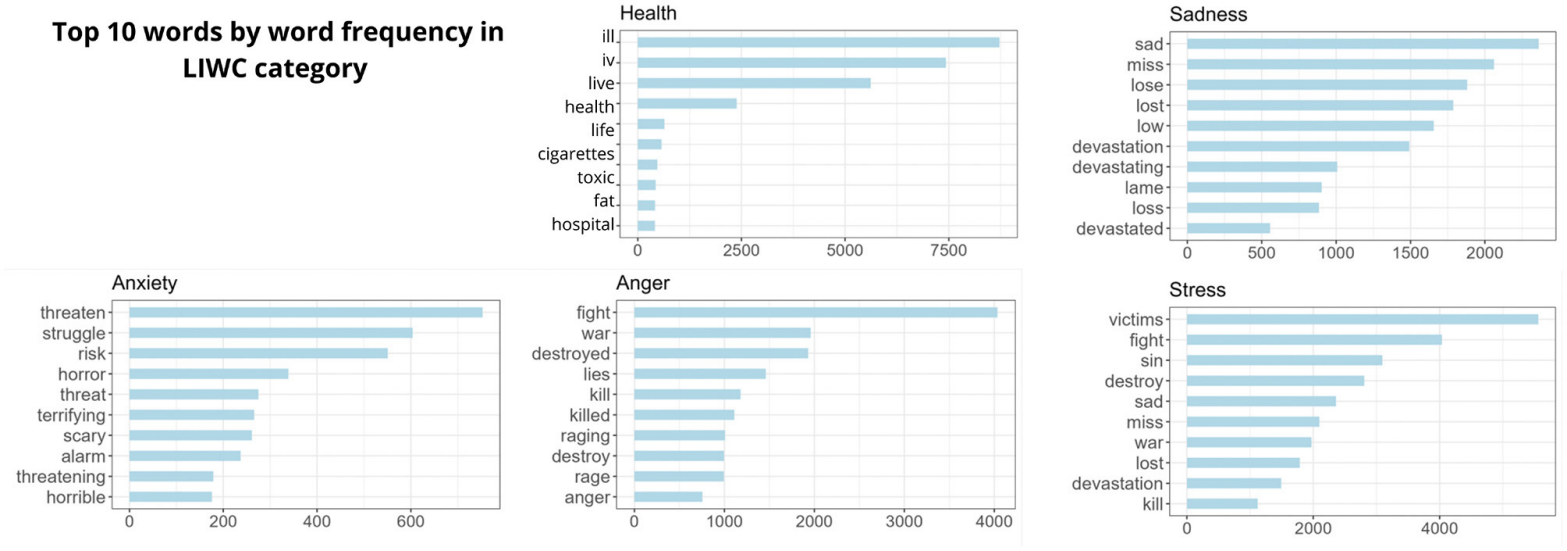
Ten most frequently used words associated with each LIWC category.

Figure 5A illustrates the distribution of tweets among different emotional categories and Figure 6 displays the ten most common words used within each category. These results offer insights into prevalent psychological and emotional themes observed throughout the study period. Leveraging the LIWC dictionary, we identified frequently occurring emotional words in tweets related to the Tubbs Fire, with a focus on categories such as anxiety, anger, sadness, stress, and health (Figure 6).

It is important to note that within the LIWC analyses, a single tweet may fall into multiple emotional categories. These findings indicate that negative emotions constitute 43.0% and positive emotions 23.1% of all tweets. Negative emotions could be associated with messages conveying a sense of distress, sorrow, discontent, or unease. These emotions are frequently conveyed through the language used, including expressions of sadness, frustration, disappointment, or concern. Conversely, “Pos Emotion” constitutes 23.1% of the total, indicating a substantial presence of positive emotional expressions among users. These sentiments could include hope, support, and optimism, possibly reflecting both resilience and communal solidarity in response to the wildfire event.

The category of “Stress” accounts for 36.3% of the total tweets, underscoring the significant psychological strain experienced by individuals during the wildfire event. The high occurrence of stress-related discussions underscores the emotional impact and the potentially distressing nature of such natural disasters like wildfires. The *sadness* and *stress* categories share several words (Figure 6). The vocabulary reflects shared grief and sorrow over what was lost in the wildfires. “Victims”, “sad,” and “miss” may evoke empathy and concern for those directly affected by the fire. “Fight” and “war” represent the struggle to contend with the fire and its aftermath. Strong words like “sin”, “destroy,” and “kill”, “devastation/devastated” and “lose/lost” could indicate stress over the loss of life caused by the fire, whether human or animal. “Low” captures the diminished mood and trauma endured by survivors. “Lame” suggests frustration regarding the uncontrollable situation (48, 49).

Furthermore, the categories of “Anger”, “Health”, “Sadness”, and “Anxiety” constitute 15.3%, 7.9%, 12.9%, and 5.9% respectively. The differing proportions within these categories emphasize the diverse range of emotions individuals go through, including feelings of anger, health and emotional wellbeing concerns, expressions of sadness, and conversations about anxiety. For a selection of messages shared during the wildfire event, please refer to Table 2.

**Table 2:** Example of the tweets shared during the wildfire event.

**Tweet**	**Emotion**
“My heart goes out to these families, my Sonoma County neighbors, as they view their beautiful community the fires laid bare. I mourn with you & support you @SenKamalaHarris @SenFeinstein #santarosafire #FridayFeeling #positivelykind #CALFIRE”	Supportive
“All the brave men and women out there are doing such a great job with containing the fires! You make us proud! @CAL_FIRE #SonomaStrong”	Positive
“People walk around Petaluma wearing masks because the smoke in the air is so bad that it's worse than smoking an entire pack of cigarettes.”	Health
“Our story last night from Napa Valley. Sad to see wine country in such a state of disaster.”	Sadness
“My family is devastated and anxious affected by this horrible #sonoma #napa FIRE ..it's been a…”	Devastated, Anxious
“Still 0% contained. Have to go to work tomorrow which is in the thick of where the fire is. 10 dead. This shit sucks”	Anxiety, Negative
“Sitting at home and feeling hopeless is really shitty.”– a cellarmaster in Napa talks about life after fire.”	Negative
“ Oh hiii California is on fire but I'm safe but I'm dead tired and my anxiety is keeping me awake!!!”	Anxiety, Negative
“#CaliforniaWildfires Depression and Anxiety are so bad now because of this fire storm. I keep hearing sirens.”	Depression, Anxiety
“I'm so stressed out. Fire in Santa Rosa. Fire in Anaheim. Idiots hitting my car. My job is so demanding. So much shit on my mind.”	Stressed, Negative
“Ach! Too sad. California wildfires kill 21 including elderly couple Find #California air quality index via zip code below. These #wildfires are devastating. So sad for those affected.”	Sad, Devastating
“The fire in Napa continues to grow.. More people have died. It's getting so scary.”	Negative
“The air quality is so bad in San Jose. The poor people of Napa/Sonoma w/the 6 fires raging. We smell it all the way down here.”	Negative
“ The fire smell from Napa is irritating my nose so bad”	Negative
“Just spoke to an older man that was a half mile away from where the fire started in Santa Rosa and just the sadness and shakiness in his voice kills me. Being here helping out has put a lot into perspective... ”	Sadness
“Very sad! Calistoga a ghost town after mandatory evacuation order...... #Calistoga #TubbsFire #NapaFire”	Sad, Evacuation
“I feel sad for all the people in Napa who are losing their houses and everything they have. It makes me so sad”	Sad, Negative
“Brutal 2017 #wildfire season is stressing state + federal agencies that pay for army required to fight fires.”	Stress
“@JuddLegum Trump is disrespecting the flag by ignoring Americans who are victims of California fire disaster.#NapaFire”	Anger
“Having lived through this emergency and still living it 8 days later, this is outrageous. Last night a fire fighter died, this morning one fell off a 50 foot cliff and has yet to be rescued. #Firestorm”	Stress, Anger
“People: This is likely the worst fire season in California history and some of you are still out jogging with your dogs in this air with no respirators...please have some situational awareness, the air is very bad for you and them right now.”	Stress
“@realDonaldTrump In case you aren't aware, California is part of the US and the entire star is on fire. Devastation everywhere! Can you address this! #moron”	Anger
“13 dead now in the Santa Rosa fire...will this horror ever end?!”	Sadness
“The smoke and ash and whatever else is in the air is making me nauseous as fuck. This is horrible and scary.”	Anxiety, Sadness, Fear

The *anger* vocabulary reflects frustration and conflict stemming from the wildfire's extensive impacts. Words like “fight”, “war”, “destroyed”, and “destroy” convey a sense of struggle against the fire's damage. “Lies” implies betrayal, potentially tied to misinformation. Strong language, including “killed” and “rage,” shows anger about lives lost and overall devastation.

The language in the *anxiety* category conveys a shared sense of distress, uncertainty, and vigilance in response to the wildfire's dangers. Words like “threaten”, “threatening,” and “risk” signal perceptions of harm, unpredictability, and vulnerability. “Struggle” represents the challenges faced in evacuating or finding shelter amidst the crisis. Intense terms like “horror”, “terrifying”, “scary”, and “horrible” express the profound fear triggered by the fire's destructive force. References to being “alarmed” capture a state of heightened alertness and need for urgent action. The vocabulary reflects the reality of living through an environmental catastrophe, with lingering uncertainty over the potential for loss and harm (50).

The *health* category provided insights into both the physical and psychological aspects, with words like “ill”, “live”, “health”, and “hospital” highlighting the impact on wellbeing and medical concerns. In contrast, terms such as “toxic” and “cigarettes” reflected participants' concerns about the smoke's toxicity, including comparisons to the harm caused by cigarettes. Moreover, these terms hinted at discussions regarding potential causes of the fire.

### 3.3 Most relevant topics during the Tubbs Fire period

We employed topic modeling to uncover the primary themes related to mental health, emotional wellbeing, and wildfires. Subsequently, the results underwent qualitative coding focusing on the top eight most relevant topics. The default model presented a range from 10 to 100 topics using default parameters in LDA models. The highest coherence score is approximately 0.43, achieved with a topic number of 60. The process of hyperparameter optimization unveiled that an optimal α value of “asymmetric” in nature, along with an optimal β value of 0.235, coupled with an optimal number of topics set at 88, notably amplified the quality of topics generated by the LDA model. When comparing the optimized LDA model with the default model, we observed a 7% increase in coherence, indicating an enhanced coherence score and topic interpretability.

#### 3.3.1 Topic interpretability

Eight topics related to wildfires, mental health, and wellbeing have been identified. Topics containing words related to different themes were excluded. It is important to note that the numbers assigned to these topics serve solely for identification purposes and do not reflect their relevance. The topics were labeled based on their defining words and the content found within tweets. This process entailed filtering tweets containing these keywords in an effort to identify the main topics. Additionally, we cross-referenced them with relevant news articles (48–52), contributing to the interpretation of the identified topics. See Table 3 for a summary of the eight topics related to the themes we are interested in.

**Table 3:** Summary of the eight topics most related with wildfire and wellbeing.

**Topic #**	**Words**	**Label**	**%**
1	fire, angry, say, California, smoke, high, wildfire, long, deadly, close	Emotional reactions	56.60%
2	smoke, day, weed, cigarette, bore, depression, hand, fall, second, part	Mental wellbeing and coping mechanisms	67.50%
3	anxiety, show, fan, heat, wildfire, month, california, insomnia, image, hue	Anxiety and media influence	44.80%
4	air, session, check, problem, quality, isolation, poor, cracker, suffocate, arm	Air quality and concerns	32.10%
5	man, tired, everyone, hope, service, tense, firework, survivor, smh, warn	Emotional exhaustion and hope	48.30%
6	watch, flame, depression, fully, race, friendly, condition, private, binge, period	Observing flames and emotional impact	35.80%
7	fuck, scary, hate, book, write, toll, class, weekend, fire, gun	Negative emotions	40.70%
8	trump, match, folk, spain, spend, shin, kitchen, likely, opinion, monday	Public criticism of Donald Trump's handling	29.80%

The percentage represents the contribution for each topic based on the word coefficients.

All topics share a common focus on the emotional and psychological impact of wildfires on individuals and communities. They collectively reflect the distress, anxiety, and challenges experienced in the context of wildfires. The emotional toll, anxiety, and distress caused by the wildfires are recurring themes across these topics. However, each topic also presents distinct nuances and focuses:

**Topic 1: Emotional reactions** focuses on the feelings of anger and frustration due to the severity and impact of wildfires. It includes terms like “fire”, “angry”, “California”, and “wildfire”, reflecting discussions about the emotional responses to the California wildfires. The inclusion of words such as “smoke”, “high”, and “close” suggests conversations about the intensity and immediate danger of wildfires, further amplifying the feelings of anger associated with these events.

**Topic 2: Mental wellbeing and coping mechanisms** explores the emotional impact of wildfires, focusing on the monotony of recovery efforts, the sadness of loss, and the urgency of decision-making. It highlights terms like “bore” and “depression”, reflecting the emotional strain and sadness from the repetitive nature of recovery and loss. The term “cigarette” could imply the harm from smoke, akin to cigarette smoke, or a potential fire cause. “Second” underscores the need for quick decisions and actions due to rapidly spreading wildfires. “Hand” symbolizes collective efforts and support needed to tackle the fire's challenges. “Fall” likely refers to California's wildfire season.

**Topic 3: Anxiety and media influence** delves into the emotional experience of anxiety triggered by wildfires and heat. It highlights the role of media coverage, represented by words like “show” and “image”. The term “month” suggests discussions about the prolonged duration of the disaster. Additionally, the mention of “insomnia” indicates conversations about the impact of wildfires on individuals mental states and sleep patterns.

**Topic 4: Air quality and concerns** addresses conversations related to the quality of the air and broader environmental concerns, including the effects of poor air quality on personal wellbeing and health, and strategies to alleviate discomfort caused by air quality problems. Terms such as “air”, “quality”, and “problem” suggest a focus on issues related to air pollution and its potential impacts. The topic also delves into the personal effects of poor air quality, as indicated by terms like “isolation” and “suffocate”. The inclusion of terms like “session” and “cracker” could point to discussions about strategies to alleviate discomfort caused by air quality issues.

**Topic 5: Emotional exhaustion and hope** underscores feelings of fatigue, tension, and potential frustration among individuals. Participants in these discussions might be expressing feelings of exhaustion in response to a challenging or stressful situation, such as monitoring a wildfire. It captures words such as “man”, “tired”, “everyone”, “tense”, “smh” (shaking my head, often used to express frustration), and “firework”. However, amidst the expressions of exhaustion and tension, there's a contrasting sentiment of hope and resilience, as indicated by words like “hope” and “survivor”.

**Topic 6: Observing flames and emotional impact** involves discussions about observing or monitoring a fire or flame, as suggested by the terms “watch” and “flame”. The term “depression” points to a potential emotional impact, emphasizing the distressing nature of wildfires and their effects on individuals and communities. This suggests that the topic also explores the emotional responses to such challenging situations. The word “condition” probably refers to the overall state of affairs, highlighting the critical conditions and challenging circumstances posed by wildfires. Lastly, the term “binge” could imply an intense or sustained period of activity or impact. This might relate to the continuous challenges posed by wildfires, suggesting a prolonged period of dealing with these distressing events.

**Topic 7: Negative emotions** signifies the presence of intense negative feelings and emotions, disruptions and concerns related to the situation, and the emotional toll that certain experiences take on individuals, potentially impacting their wellbeing. The terms “fuck”, “scary”, and “hate” strongly signify the presence of intense negative feelings and emotions. The term “class” may be linked to the cancellation of classes as a result of the Napa fire's proximity. This suggests potential disruptions and concerns related to the situation. The use of the word “write” may be connected with the act of written communication, particularly in expressing emotions and concerns during difficult times. The reference to the “book” may have a religious or spiritual connotation, possibly highlighting reflections on life, death, and the impact of the wildfires. The term “fire” in this context could refer to literal wildfires and metaphorical situations generating negative emotions. Additionally, terms like “weekend” and “toll” might highlight the emotional toll that certain experiences take on individuals, potentially impacting their wellbeing.

**Topic 8: Public criticism of Donald Trump's handling**. In summary, the identified words align with a topic centered on public criticism of Donald Trump's handling of the California wildfires. The terms “match”, “folk”, “spend”, “likely”, “opinion”, and “Monday” contribute to the overall narrative, reflecting discussions on government response, public sentiment, and the urgency of the situation. The mention of “kitchen” could be a reference to the reported incident of a fire burning in the kitchen area of a restaurant.

### 3.4 Awareness of the mental health challenges

In examining the keywords within the topics, we discovered messages that advocate for supporting the victims, underscoring a sense of community solidarity:

- “*Info on how to donate or support all affected by NorCal fires including our first responders. Please share to help*”.
- “*California American Water &amp; American Water Charitable Foundation Provide Support to California Wildfire Relief…*”
- “*HELP me raise $10k to Support The Northern California Wine Country Fire Efforts @RedCross donate*”.

These messages not only express a call to action but also underscore the collective effort and community solidarity in supporting those affected by the wildfires.

Additionally, there were messages offering support for mental health:

- “*The Disaster Distress Helpline. 1-800-985-5990 toll-free or text*”
- “@*SRCSsup: School psychologists, counselors &amp; other mental health professionals available to discuss trauma of fire*.”
- “*Mental health resources for wildfire disasters:* https://t.co/EDx7j71pLb”

These messages reflect a compassionate response, indicating an awareness of the mental health challenges posed by the wildfires and a proactive commitment to offering the necessary support.

## 4 Discussion

The main goal of this research was to identify emotional wellbeing and mental health indicators in individuals who were impacted by the Tubbs Fire, which was one of the most devastating wildfires in California in 2017. The study utilized Twitter data and applied various analytical methods to achieve this objective. The methodologies used included sentiment analysis, LIWC analysis, identification of frequently occurring words (53), and topic modeling through LDA.

The results illustrate heightened user engagement patterns on Twitter, with increased activity observed during nighttime and early morning, especially at the onset of wildfire incidents. Several factors could influence this temporal pattern. For instance, users might be more active in the afternoon, potentially during work or school breaks, night or early morning may appeal to individuals dealing with sleeplessness. Past research on depression has suggested a link between increased activity during these hours and depressive symptoms (54). This suggests a potential connection between increased Twitter activity, concerns related to wildfires, and more extensive psychological and behavioral factors. Moreover, the heightened vigilance during the evening and night, when wildfires are more noticeable, might contribute to the amplified online activity.

This observation could provide a strategic opportunity for public health authorities to implement focused media campaigns to raise awareness about mental health and emotional wellbeing during wildfires. By aligning these efforts to match the nighttime and early morning hours, authorities can efficiently reach and support individuals when they are most actively discussing and experiencing the emotional challenges associated with wildfire events.

Employing LIWC categories in the analysis facilitated the revelation of discrete emotional dimensions and identifying keywords encapsulating these sentiments, enhancing the depth of understanding regarding human experiences amidst crises. Examination of emotional categories, including “Anger”, “Health”, “Sadness”, “Anxiety”, “Stress”, and “Positive/Negative emotions”, illustrates the complexity of emotions experienced by individuals. By analyzing the emotional responses within the dataset, it becomes evident that Twitter users engaged in discussions that primarily conveyed negative emotions, as indicated by the significant presence of the “Neg Emotion” category, comprising 43.0% of total tweets. Conversely, 23.1% of tweets fell into the “Pos Emotion” category, reflecting a substantial presence of positive emotional expressions. These positive sentiments may encompass hope, support, and optimism, indicative of resilience and communal solidarity in response to the wildfire event. However, the category of “Stress” accounted for 36.3% of total tweets, underscoring the significant psychological strain experienced by individuals during the wildfire event. The varying proportions within these categories reveal a spectrum of emotional reactions, encompassing anger, concerns about health and emotional wellbeing, expressions of sadness, and discussions revolving around anxiety.

This linguistic analysis sheds light on the emotional landscape of individuals engaging with the Tubbs wildfire on social media, aligning with previous research emphasizing language's role in revealing collective responses to environmental disasters (55–58). The distribution of words associated with these emotional categories further provides insights into the prevailing sentiments, providing potential clues about their mental wellbeing and emotional responses during the wildfire event.

Identifying keywords and key topics associated with wellbeing and mental health serves as a tool for connecting users expressing distress, anxiety, or depression with appropriate resources, such as helplines or local mental health services. This information not only streamlines the provision of timely assistance but also provides insights into what resources must be developed to help during crises, ensuring that individuals facing mental health challenges receive the urgent support they require. Furthermore, this information allows for the customization of social media campaigns to effectively address the most prevalent issues highlighted through this kind of analysis. However, as language and how people express themselves evolve, it becomes important to stay informed about available resources and continuously update the keywords used to identify users needing support.

The topic modeling has revealed eight distinct topics that offer insights into potential avenues for intervention by policymakers during wildfire events. The general ideas of the topic provide information about (1) emotional reactions, mental wellbeing, and coping strategies during wildfire events: people express sadness, insomnia, anxiety, and depression for the situation; (2) emotional exhaustion and support: individuals convey their support for the victims and express gratitude for the efforts undertaken by the firefighters also expressing fatigue and weariness; (3) air quality: concerns were raised regarding the health impact of smoke, drawing parallels to the effects caused by cigarettes. Simultaneously, there was speculation about a cigarette being the cause of the fire. However, after an investigation lasting over a year, the California Department of Forestry and Fire Protection (Cal Fire) determined that the Tubbs Fire was caused by a private electrical system adjacent to a residential structure (59), and 4) Public criticism of Donald Trump's handling. During that period, Donald Trump held the presidency, and there was considerable controversy surrounding his position on forest management (60).

The analysis of keywords within the discussed topics also unveiled a notable display of community support and solidarity during the wildfires. Messages urging support for the victims, exemplified by calls to donate, assistance for first responders, and fundraising efforts, underscored a collective determination to alleviate the fires' impact. These messages reflect a call to action and demonstrate the community's resilience and unity in times of crisis. Additionally, the discussions exhibited a noteworthy focus on mental health, with messages providing resources and helpline information for those grappling with the emotional toll of the wildfires. This dual emphasis on material support for victims and mental health resources highlights the multifaceted response of the community, showcasing a compassionate and proactive approach to addressing the challenges posed by wildfires.

We offer a potential interpretation of the identified topics, and feasible interventions according to the findings. However, it is crucial to recognize that the insights extracted from the topic modeling analysis may not encompass the full spectrum of relevant factors. Consequently, further research initiatives are necessary for a better understanding of the intricate elements influencing wildfires' impact on individuals and communities. Furthermore, various algorithms, including LDA and BERT, are available to explore topic modeling in the analysis of Twitter data during crises (61). Latent Dirichlet Allocation (LDA) may perform better than BERT when faced with rapid responses when identifying relevant topics. LDA's advantages include superior speed and scalability, enabling efficient processing of large volumes of Twitter data in real-time or near-real-time. Moreover, LDA's interpretability facilitates the identification of prevalent topics and critical emerging themes, aiding in swift decision-making and stakeholder communication. Its simplicity further enhances its utility. LDA's lower resource requirements and reduced dependence on labeled training data make it a practical choice, particularly in scenarios where computational resources or labeled data for fine-tuning BERT are limited. Therefore, LDA emerges as a well-suited tool for promptly identifying relevant crisis topics. Conversely, BERT may be more useful in contexts where deeper understanding and ample computational resources are available (62, 63).

**Figure 7:**
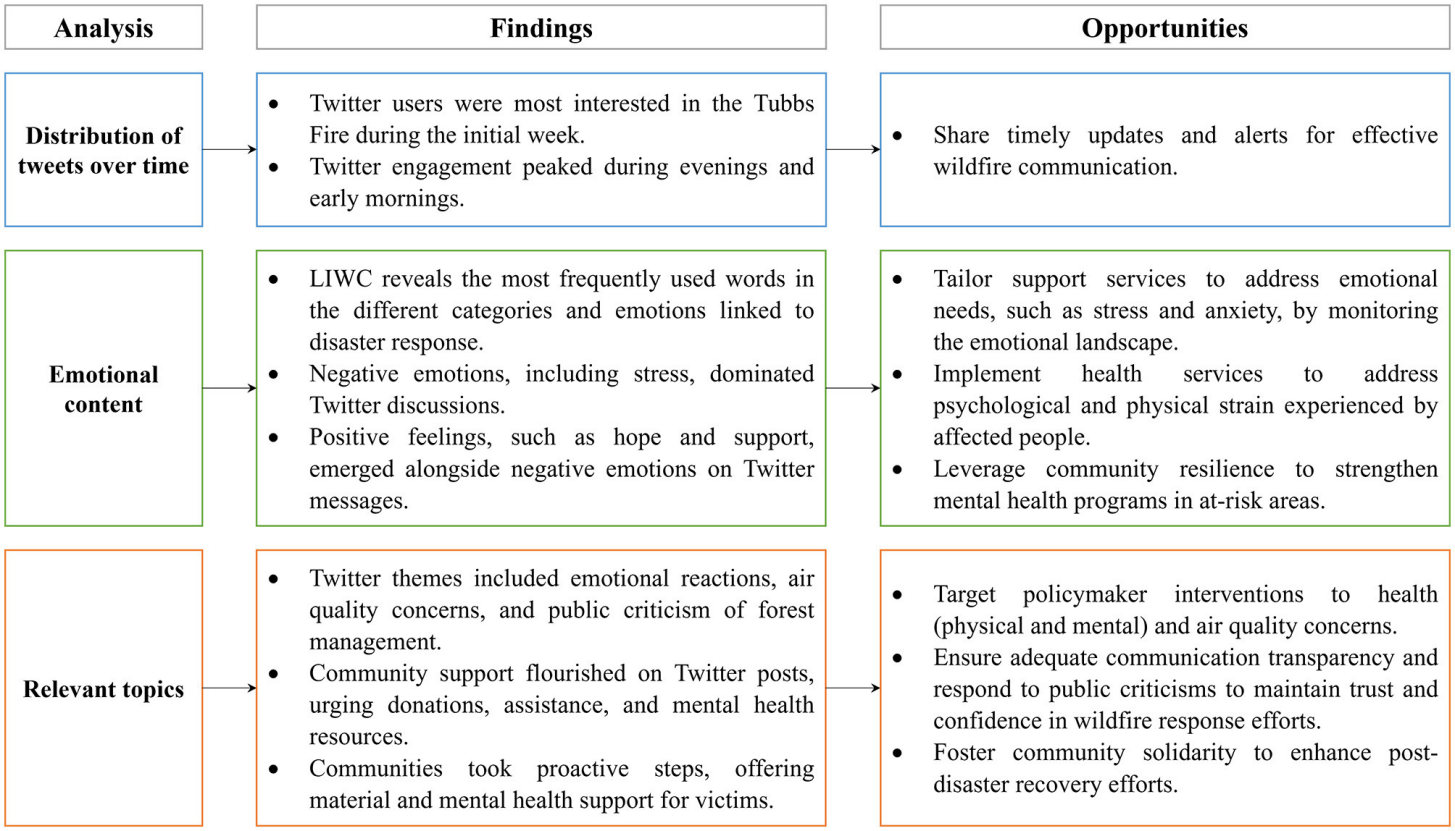
Summarized approaches to mitigate the adverse events of wildfires reflected in the Tubbs Fire-related posts on Twitter.

The findings of this study can serve as a signpost for identifying key topics or initiating further investigations. Social media platforms can serve as valuable tools for real-time data collection through user-generated content and by employing targeted surveys (64), which can facilitate enhanced resource allocation strategies. Obtaining a comprehensive understanding of user dynamics during a wildfire event has the potential to refine and optimize intervention efforts. In addition, leveraging social media analysis to discern sentiment might provide further guidance for policy-making (65, 66), allowing informed decisions that align with the emotional and psychological wellbeing of individuals impacted by wildfires. The study allowed us to formulate general proposals for reducing the adverse consequences of wildfires, mainly those related to mental health and wellbeing. Accordingly, Figure 7 summarizes the approaches to mitigate the emotional distress and discomfort reflected in the Tubbs fire-related posts on Twitter.

Previous studies have leveraged Twitter data to extract and analyze emerging topics of concern during and after disasters. For example, (28) conducted a study using Twitter data to identify emerging topics of concern during the Camp Fire. Similarly, Zhai (67) and Mendon (68) explored Twitter data to extract and examine disaster-related topics. Other works focus on tweets related to the 2019–2020 Australian bushfires. These include Gardiner et al. (69) seeking to understand which communities and stakeholders generate and exchange information about disasters caused by natural hazards, and Zander et al. (70) using tweets collected through hashtags to discover trends in the most affected areas. In addition, crisis events such as the COVID-19 pandemic were used to understand the sentiment in real-time. For example Xue et al. (71), explored public discourse and sentiment during the pandemic using LDA to model topics on Twitter. Ng et al. (65) analyzed emotional reactions to perceived invidious policies or safety and effectiveness concerns related to COVID-19 vaccines, and Ng et al. (66) investigated public messages about loneliness through unsupervised machine learning analysis of posts made by organizations on Twitter. Nevertheless, in contrast to prior research that compared shared concerns across different social media platforms, this study focuses on identifying signals about mental health and emotional wellbeing during the Tubbs Fire in California using a range of available analysis. The analysis includes sentiment analysis, topic modeling, and LWIC to uncover the underlying patterns and themes in the Twitter discourse.

## 5 Limitations and threats to validity

While providing insights into mental health and emotional wellbeing during wildfire-related events, this study acknowledges several limitations. One noteworthy limitation pertains to the data collection. As we prepared this paper, Twitter's data access policies changed, posing challenges in extending our analysis to real-time surveillance utilizing Twitter data. Nonetheless, the insights gleaned from our analyses remain applicable and transferrable to other forms of social media data, ensuring the generalizability of our findings beyond the confines of Twitter.

Moreover, beyond the limitations inherent in the data collection methods and the scope of the dataset, there are also considerations related to the demographic characteristics of Twitter users, which may be different from the general population. They tend to be younger, more urban, and more educated than the average population (72, 73). This could lead to bias in the findings.

The applicability of Tubbs fire Twitter data to other fire events hinges on factors like the fire's specifics, community characteristics, Twitter usage patterns, and the study's time frame. Factors such as the severity of the fire, the level of preparedness of the community, the resources available for recovery, and the specific demographic and socioeconomic characteristics of the affected individuals can all influence mental health outcomes and the manifestation of these outcomes on social media. Additionally, not everyone uses Twitter, and usage varies among those who do. Lastly, the time frame of the study could also influence its generalizability. Mental health impacts can evolve, and a study capturing data from a specific period may not fully encapsulate these longitudinal effects (51, 52).

By focusing solely on English, the study's language filtration has excluded non-English-speaking communities in California, including Spanish-speaking individuals. This group represents one of the state's largest minorities (74, 75) and is among the most susceptible to the enduring impacts of natural disasters like wildfires (76). Additionally, a limitation exists in the terms used for data retrieval, as the emotional categories, although based on previous research, might not fully cover the range of sentiments and emotions expressed, especially those conveyed through colloquial expressions. Finally, it is essential to acknowledge that the analysis in this study is primarily descriptive and serves as an initial foray into understanding emotional responses during wildfire events. Subsequent research can delve deeper into these themes, offering more comprehensive insights into the complex interplay of emotions during such crises.

Additionally, unsupervised machine learning approaches also have inherent limitations, particularly concerning interpreting the intent behind posts and the potential for misclassification. Unsupervised learning techniques, such as sentiment analysis and topic modeling, rely on identifying patterns within data without explicit guidance, which can present challenges in accurately deciphering the nuanced context or subtle nuances in language, including sarcasm or ambiguity. Additionally, there exists a notable risk of misclassification, where algorithms may inaccurately categorize data due to biases in training data or limitations in generalization abilities. Traditional metrics for model performance may not be directly applicable, making it challenging to objectively assess the quality of clustering or topic modeling results. Despite these challenges, during a natural disaster like a wildfire, utilizing social networks alongside the methodologies discussed herein offers an initial avenue to identify prevalent discussion topics among individuals potentially linked to the impact on mental health and wellbeing. Nonetheless, for a more comprehensive analysis and comprehension, it becomes imperative to delve into alternative tools and forms of analysis, enabling a deeper exploration of the complexities underlying such discourse.

While Latent Dirichlet Allocation (LDA) presents numerous advantages in topic modeling, it possesses limitations in comprehending intricate contexts and capturing subtle nuances within text data. When the complexity of the data surpasses LDA's capabilities, BERT emerges as a more suitable alternative. BERT's deep learning architecture lets it quickly grasp semantic relationships and contextual nuances, leading to more precise topic identification in challenging datasets. Therefore, when confronted with text data requiring a deeper understanding of context, BERT may offer superior performance compared to LDA (61).

## 6 Conclusion and future work

This study proposes a different approach to rapidly gain insights into user mental states and calls for action, especially during environmental crises like wildfires, in contrast to traditional surveys.

The change in Twitter APIs and their unavailability pose challenges in accessing real-time Twitter data, so alternative data collection methods or adjustments are necessary to address these changes. Thus, the proposed methodology is adaptable to other natural disasters like earthquakes or floods and can be implemented on various social media platforms, including established ones like Reddit or emerging platforms like Thread.

Policymakers can leverage this insight to develop targeted mental health support programs that assist individuals facing emotional challenges. They can promote programs such as support networks, counseling services, and community-building activities to aid individuals and communities in coping with the stress and fatigue commonly associated with wildfires (16), and prioritize comprehensive air quality management measures, including monitoring and enhancing air quality and providing guidance on indoor air purification techniques to alleviate the discomfort experienced by individuals affected by wildfires (20).

Future work involves identifying additional sources of social media data beyond Twitter. By incorporating data from platforms such as Facebook, Instagram, and Reddit, we would like to create a system for real-time monitoring. This expansion will enable us to capture a broader spectrum of user-generated content, including images, videos, and text-based posts, thereby enhancing the effectiveness and scope of our monitoring efforts. Additionally, integrating other advanced data analytics and natural language processing techniques will facilitate the extraction of valuable insights from the collected data, enabling us to identify emerging trends, sentiments, and relevant information about wildfires, air quality, and public health concerns.

Furthermore, the inclusion of tweet geolocation within the analysis should be considered, using metadata from the APIs of the chosen social media network, lexicons, or through the development of user networks. Researchers can concentrate on validating this information correctly, as this would enrich the analysis of social media data. Incorporating geolocation would provide a more complete and accurate perspective of the geographic distribution of online conversations, which could have broad implications for decision-making and understanding social behaviors.

## Data availability statement

The original contributions presented in the study are included in the article/Supplementary material, further inquiries can be directed to the corresponding author.

## Author contributions

YG: Conceptualization, Data curation, Formal analysis, Investigation, Methodology, Visualization, Writing – original draft. MV-P: Conceptualization, Data curation, Investigation, Methodology, Writing – review & editing. KL: Formal analysis, Methodology, Writing – original draft. XHT: Conceptualization, Funding acquisition, Writing – review & editing. LT: Conceptualization, Methodology, Supervision, Writing – review & editing. MD-T: Conceptualization, Methodology, Writing – review & editing. JM-L: Conceptualization, Methodology, Writing – review & editing. MN: Conceptualization, Funding acquisition, Methodology, Supervision, Writing – review & editing.

## Footnotes

^1^ https://github.com/yurygarcia26/Wildfires_MentalHealth

^2^ https://ieee-dataport.org/documents/twitter-tubbs-fire-dataset

^3^ https://pypi.org/project/vaderSentiment/
