# Supplementary material for: Wildfires and social media discourse: exploring mental health and emotional wellbeing through Twitter
Source: Front Public Health. 2024 Apr 12;12:1349609. doi: 10.3389/fpubh.2024.1349609 (PMC11046489; doi:10.3389/fpubh.2024.1349609)
Supplement: Supplementary file 1 [file Data_Sheet_1.PDF]

Supplement Material: Wildfires and Social Media Discourse: Exploring Mental Health and Emotional Well-Being Through Twitter

Yury E. García Miryam Elizabeth Villa-Pérez Kuang Li Xiao Hui Tai, Luis A. Trejo, Maria L. Daza–Torres, J. Cricelio Montesinos-López, and Miriam Nuño

Keywords used during the data collection process

Tubbs Fire (Octobre 8-31 2017)
Period to download tweets: Septiembre 8 - Noviembre 30 Coordinates 38.60895°N 122.62879°W
Most affected communities: Santa Rosa, Napa, Sonoma, Calistoga, Petaluma impacted by smoke: Sonoma, Lake, Mendocino, Solano

The Tubbs Fire was a devastating wildfire that occurred in California in October 2017. Some of the most popular hashtags used during the Tubbs Fire on Twitter were:

#TubbsFire, #NapaFire, #SantaRosaFire, #CaliforniaWildfires, #SonomaStrong, #PrayForCalifornia, #WineCountryFires, #FireStorm, #NorthBayFires, #SFBayFires, #SonomaFire, #CaliforniaWildfires, #NorCalFires, #airquality, #Smokeintheair, #smokeyair

query = """-RT (#TubbsFire OR wildfire OR smoke OR #NapaFire OR #SantaRosaFire OR #WineCountryFires OR #PrayForCalifornia OR #CaliforniaWildfires OR #NorCalFires OR #airquality OR #smokeintheair OR #smokeyair OR fire OR Nightmares OR Anxiety OR Depressio OR Panic attacks OR Survivor guilt OR #PTSD OR PTSD OR stress OR stress OR struggle OR struggle OR sad OR #sad OR #flashbacks OR #californiafires OR #evacuation OR #disaster OR #fatigue OR #smokeinhalation OR #respiratoryproblems OR #asthma OR #COPD OR #lunghealth OR #burningeyes OR #sorethroat OR #headaches OR #fatigue OR #stress OR coughing OR wheezing OR breath OR asthma OR bronchitis OR (Eye irritation) OR (nose irritation) OR congestion OR irritation OR Headaches OR fatigue OR itching OR irritation OR redness OR inflammation OR dryness OR flakiness OR discomfort OR rashes OR hives OR "I was diagnosed" OR "I've been diagnosed" OR "I have been diagnosed" OR "I'm diagnosed") pointradius:[-122.62879 38.60895 40km] lang:en -is:retweet"""

Tweets Examples

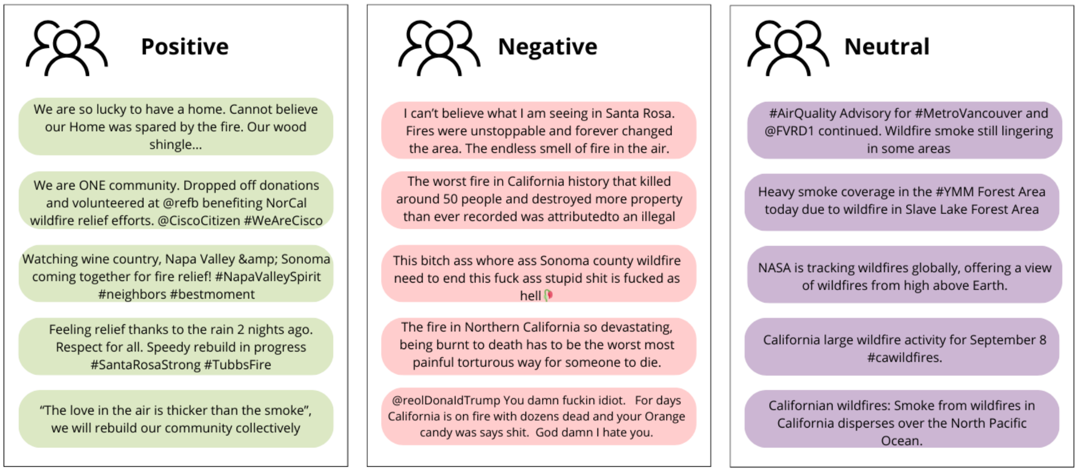

Figure S1. Examples of tweets categorized as Positive, Negative, or Neutral according to the VADER algorithm.
